# The Healthy Children, Strong Families intervention promotes improvements in nutrition, activity and body weight in American Indian families with young children

**DOI:** 10.1017/S1368980016001014

**Published:** 2016-05-23

**Authors:** Emily J Tomayko, Ronald J Prince, Kate A Cronin, Alexandra K Adams

**Affiliations:** 1 Department of Nutritional Sciences, University of Wisconsin, College of Agricultural & Life Sciences, Madison, WI, USA; 2 Department of Family Medicine, University of Wisconsin School of Medicine and Public Health, 1100 Delaplaine Court, Madison, WI 53715, USA

**Keywords:** Home-based intervention, Nutrition, Physical activity, Paediatric obesity, Early childhood, American Indian, Community-based participatory research, Family

## Abstract

**Objective:**

American Indian children of pre-school age have disproportionally high obesity rates and consequent risk for related diseases. Healthy Children, Strong Families was a family-based randomized trial assessing the efficacy of an obesity prevention toolkit delivered by a mentor *v*. mailed delivery that was designed and administered using community-based participatory research approaches.

**Design:**

During Year 1, twelve healthy behaviour toolkit lessons were delivered by either a community-based home mentor or monthly mailings. Primary outcomes were child BMI percentile, child BMI *Z*-score and adult BMI. Secondary outcomes included fruit/vegetable consumption, sugar consumption, television watching, physical activity, adult health-related self-efficacy and perceived health status. During a maintenance year, home-mentored families had access to monthly support groups and all families received monthly newsletters.

**Setting:**

Family homes in four tribal communities, Wisconsin, USA.

**Subjects:**

Adult and child (2–5-year-olds) dyads (*n* 150).

**Results:**

No significant effect of the mentored *v*. mailed intervention delivery was found; however, significant improvements were noted in both groups exposed to the toolkit. Obese child participants showed a reduction in BMI percentile at Year 1 that continued through Year 2 (*P*<0·05); no change in adult BMI was observed. Child fruit/vegetable consumption increased (*P*=0·006) and mean television watching decreased for children (*P*=0·05) and adults (*P*=0·002). Reported adult self-efficacy for health-related behaviour changes (*P*=0·006) and quality of life increased (*P*=0·02).

**Conclusions:**

Although no effect of delivery method was demonstrated, toolkit exposure positively affected adult and child health. The intervention was well received by community partners; a more comprehensive intervention is currently underway based on these findings.

Childhood obesity is experienced disproportionately by low-income and racial/ethnic minority children for multiple reasons, including environmental factors, culture, genetics, access to healthy foods and community safety^(^ ^1^ ^–^ ^3^ ^)^. In particular, American Indian (AI) children have the highest obesity rates among low-income pre-schoolers in the USA (20·8 %) and are the only group to have experienced an increase since 2003^(^ ^4^ ^)^. Increased weight in these young children persists into later life and significantly increases the risk for development of chronic diseases^(^ ^5^ ^)^. Recent evidence indicates that disparities in obesity prevalence are evident at very young ages^(^ ^6^ ^)^, suggesting the need for tailored and/or more intensive intervention strategies. However, few primary prevention studies have targeted children of pre-school age^(^ ^7^ ^)^.

Early childhood is a critical window for the development of health-related behaviours (e.g. food preferences, activity patterns) through the guidance of caregivers (i.e. parents or guardians)^(^ ^8^ ^)^. Despite recognition of the importance of the home environment in behaviour development^(^ ^9^ ^,^ ^10^ ^)^, most approaches designed for pre-school children have been administered within pre-schools or early child-care settings, where infrastructure exists to support programmatic interventions. A recent study demonstrated that involving parents/families in the research process improved health outcomes in low-income pre-schoolers^(^ ^11^ ^)^, but interventions targeting the family within the home are generally lacking^(^ ^7^ ^,^ ^12^ ^)^. This gap in the literature may be related to challenges inherent in understanding and being sensitive to differences among families, communities and cultures.

Using a community-based participatory research (CBPR) approach, we worked with four AI communities to develop the Healthy Children, Strong Families (HCSF) curriculum to promote obesity prevention among AI families with young children^(^ ^13^ ^,^ ^14^ ^)^. The aim of the present study was to test the efficacy of this obesity prevention toolkit (i.e. the HCSF curriculum), delivered either by home mentors or monthly mailings, to impact child and adult weight status, nutrition and physical activity behaviours, and self-efficacy for behaviour change in the home using a randomized trial design. The curriculum was based on the AI model of elders teaching life-skills to the next generation and was designed to reinforce the cultural values of family interaction, healthy traditional foods and activity. Moreover, the study design was based on the communities’ desire not to include a group that received no intervention. We hypothesized that the toolkit would improve weight status and health-related behaviours and the effect would be greatest for the in-home mentoring group. In alignment with CBPR principles, we sought to balance high scientific rigour with the needs and preferences of our community partners. In the present paper we describe the findings from the trial, the challenges encountered while conducting a randomized trial using community-engaged approaches in a real-world setting and how these challenges impacted the study implementation as well as data analysis and interpretation.

## Methods

### Study design

**Fig. 1:**
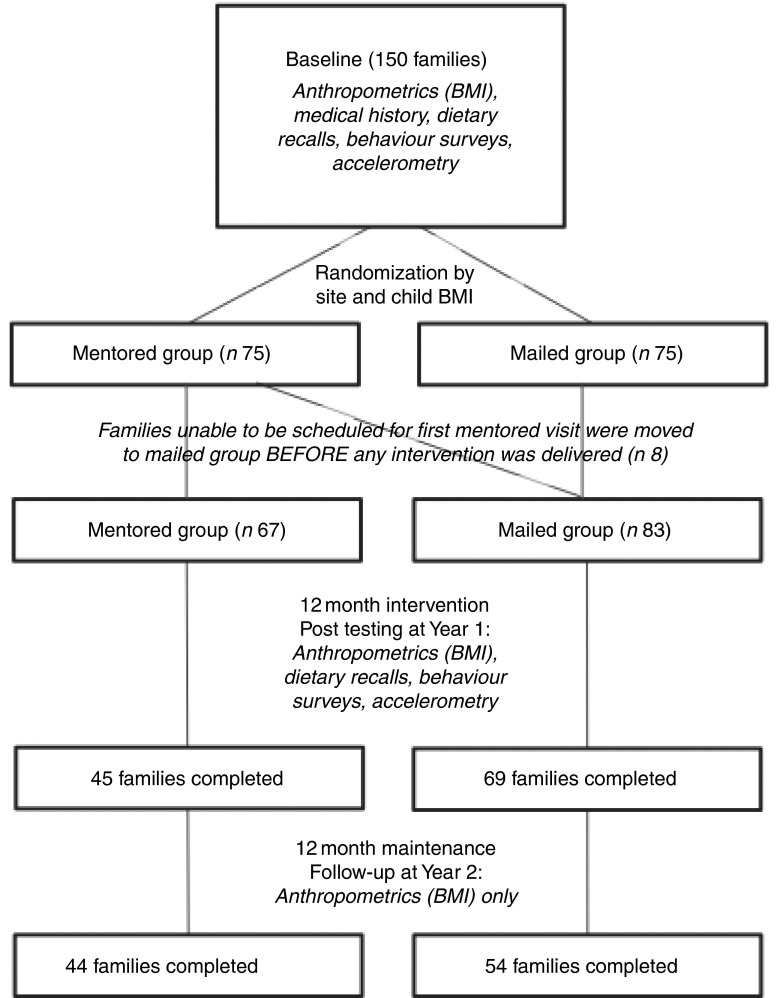
Healthy Children, Strong Families study flow diagram. ‘Family’ indicates the adult–child dyad

HCSF was a two-year, family-based, randomized trial of a healthy lifestyle toolkit delivered via two formats: in-home mentoring or by mail^(^ ^14^ ^)^. The intervention was designed with substantial community input in alignment with CBPR principles, including choosing a study name and developing the toolkit. In-home mentoring was chosen as a delivery method because many families were familiar with this approach through the Head Start programme and we believed this modality would be well accepted. It was important to the tribes to not have a group receiving no intervention; therefore, mailed delivery of the toolkit was chosen for comparison with the more time- and resource-intensive in-home delivery. The intervention was delivered to families in four Wisconsin AI tribes during Year 1, with continued support through a maintenance year (Year 2, Fig. 1). Focus group testing was performed after Year 2 to examine factors related to programme acceptance and administration. To better understand weight change over time within AI adults, an age- and gender-matched random tribal clinic sample was used as a comparison to examine changes in adult BMI, the primary outcome for adults (a comparable clinic sample for children was not available). For this clinic sample, a chart review from tribal clinic records was performed to obtain BMI on two occasions, separated by approximately 12 months, to approximate the HCSF study timeline. These BMI measures were compared with the HCSF participant BMI measures using repeated-measures ANOVA with clinic sample *v*. study sample as the between-subjects factor.

HCSF was the first randomized trial any of these tribal communities had ever undertaken and was funded as part of a larger collection of trials through a National Institutes of Health/National Heart, Lung, and Blood Institute U01 mechanism to help improve health in AI communities. Trial registration was not required at the time of study initiation.

### Study sample

A recruitment brochure was sent home with children at Head Start sites. Eligibility criteria included children 2–5 years old who lived with at least one primary caregiver (e.g. mother, father, grandmother, aunt) in a home setting and who were free of any major physical or behavioural disorders. The community partners placed a high value on inclusion; therefore, exclusion criteria were minimal. Moreover, the HCSF curriculum focuses on healthy lifestyles and no participants were excluded based on weight status. Randomization was conducted at the individual level after stratification for community and child weight status (i.e. overweight *v*. normal weight). After randomization, some families in the mentored group were unable to be scheduled for the in-home sessions. Extensive discussion with the participating communities indicated they did not want families to be removed from the study but preferred them to be switched to the mailed-only group. While not in accordance with traditional randomized trial protocols, this decision to switch the families prior to any intervention administration was supported by both the communities and the study programme officer.

### Outcome measures

Baseline measures were obtained before randomization and all primary and secondary outcome measurements (including physical measures, surveys and accelerometry) were repeated at the end of Year 1. An additional anthropometric measurement was obtained at the end of the maintenance Year 2. Medical information and family history, including basic demographic information, were collected via questionnaire at baseline only. All measurements and surveys were collected by trained study personnel at the tribal clinics, at Head Start sites or in the participants’ homes. Tribal capacity necessitated that the local site coordinator both administer the intervention and collect data from participating families. All data were mailed to the University of Wisconsin; data entry and analysis were conducted by researchers who were blinded to group assignment.

### Primary outcomes: BMI (adult) or BMI Z-score (child)

Height was measured with a portable stadiometer to the nearest 0·1 cm. An electronic scale (Tanita Body Composition Analyser, Tokyo, Japan) measured weight to the nearest 0·1 kg. All measurements were conducted without shoes and in light clothing. Child height and weight values were converted to *Z*-scores and percentiles using the parameters of the Centers for Disease Control and Prevention^(^ ^15^ ^)^. Percentiles were included because of their use in clinical settings. Overweight for children was defined as a BMI≥85th percentile and obesity as a BMI≥95th percentile^(^ ^16^ ^)^. Adult BMI (kg/m^2^) was classified according to the Centers for Disease Control and Prevention criteria^(^ ^17^ ^)^. Measured height and self-reported pre-pregnancy weight were recorded for pregnant adults.

### Secondary outcomes: nutrition and activity behaviours

Three 24 h dietary recalls were obtained (on non-consecutive days and including a weekend day) from adults for themselves and the child to assess intake. Daily servings of fruits/vegetables, sugar-sweetened drinks and candy/junk food were quantified using the Nutrition Data System for Research software 2010 (Nutrition Coordinating Center, University of Minnesota, Minneaplos, MN, USA) as previously described^(^ ^18^ ^)^. After each dietary recall, adults were asked about television (TV)/screen time use during the previous 24 h, including time the TV was on and time spent watching TV (or any screen). Screen times were totalled (i.e. minutes watching TV plus minutes on the computer) and averaged to produce an average hours per day for each adult and child for both screen time measures: time on and time watching. Physical activity was measured using Actical accelerometers (MiniMitter; Respironics Co., Bend, OR, USA) that were worn on a belt around the hip for 5–7 d each at baseline and post-testing for both adults and children. Percentage sedentary time was measured and reported because this category of activity represents the largest proportion of time for both adult and child participants (compared with vigorous, moderate and light activity).

### Secondary outcomes: psychosocial factors

The Healthy Behaviors questionnaire assessing self-perception of efficacy for health-related behaviours was administered to adults along with the SF-12, a widely used instrument for assessing self-perceived health status (including perceived Mental Health and Physical Health subscores)^(^ ^19^ ^)^.

### Intervention delivery

#### Year 1

Using a CBPR process, our University of Wisconsin research staff, University of Wisconsin Extension, academic consultants, tribal wellness staff and tribal home mentors designed toolkits consisting of twelve culturally appropriate lesson plans, activities and resources as previously described^(^ ^13^ ^)^. Each lesson addressed one of four target areas: (i) eat more fruits and vegetables; (ii) consume less soda and added sugar; (iii) become more active; and (iv) watch less TV. Families randomized to the mentored group received toolkit lessons from a trained home mentor during twelve monthly home visits (~60 min each). Home mentors were tribal members or individuals who had long-standing employment within the community; all mentors were trained to administer the intervention. Non-mentored families received toolkit lessons by mail. If at any time the home visits were unable to be scheduled or completed for participants in the mentored group, the intervention materials were provided by mail.

#### Year 2

For this maintenance year, mentored families received a monthly newsletter and participated in monthly group meetings to support behaviour changes developed during Year 1. The non-mentored families (i.e. the mailed group) received only the newsletters.

### Focus group testing

Five focus groups with study participants (twenty-five caregivers total) were conducted after Year 2. Group sessions were organized by study arm (mentored *v*. mailed) and involved recall of experiences with the intervention. One programme evaluation session was conducted with the home mentors to assess lesson content, programme administration and lessons learned.

### Statistical analysis

The study was powered to detect changes in the primary outcome measures, BMI *Z*-score (children) and BMI (adults). Repeated-measures ANOVA with study arm as the between-subjects factor was used to assess time×treatment interaction effects on all outcomes for mentored *v*. mailed toolkit delivery using intention-to-treat analyses. In addition, repeated-measures ANOVA on the combined groups was used to assess the effect of the toolkit delivered through either mechanism over time (i.e. pre- *v*. post-intervention for all study participants). Because HCSF was a healthy lifestyles trial and not a weight-loss study, we chose to also analyse adults and children by weight status. This approach was used to increase the clinical applicability and to examine if children crossed weight status. We felt this was important, as it would acceptable for a child to increase in percentile if he/she remained within normal weight status, while it would be detrimental for a child to cross from normal weight status to overweight. Conversely, it would be useful to know if children who were overweight or obese at baseline improved their weight status post-intervention. For all analyses, significance was set at *P*<0·05.

### Dropouts and multiple imputation

After randomization, participants who were unable to be scheduled for their initial mentoring visit within two months were moved to the mailed toolkit group, resulting in a higher number of participants in this group (eight families were transferred before any intervention was administered, resulting in eighty-three in the mailed-only group instead of the seventy-five expected after randomization). Participants were moved rather than dropped from the study at the request of the communities in alignment with CBPR approaches. Of the sixty-seven families who initiated the in-home mentoring programme, 67 % completed post-testing (Year 1) and 66 % completed follow-up measurements (Year 2), compared with 83 % and 66 % of the families who received mailed toolkits only. Due to the number of dropouts from both groups, multiple imputation was conducted to provide data on missing outcome variables^(^ ^20^ ^)^. Key outcome variables were examined for missingness using the Missing Values analysis in the statistical software package IBM SPSS Statistics version 22.0. Post-study (Year 1) variables were missing in approximately 30 % of cases. Based on recommendations^(^ ^21^ ^,^ ^22^ ^)^, thirty imputations were created using a multivariate normal Markov chain Monte Carlo approach. Outcomes analysed using imputed data did not differ significantly from the outcomes determined using the original data set; therefore, these imputed data are not shown.

### Community data review

As described above, community wellness staff were consulted during the data analysis process; as a group, it was decided that each community partner would have one month to review and comment on the data and any resulting manuscripts prior to publication, representing an important step in the CBPR process. Input from community partners also informed data analysis: when initial analysis revealed no differences between study arms (mailed *v*. mentored), extensive discussions with the tribes and the study programme officer informed the decision to analyse all participants as a pooled sample pre- and post-intervention. This approach allowed us to examine the efficacy of the toolkit, regardless of delivery method, to help inform any future application of the materials (similar to an approach described by Margolius *et al*.^(^ ^23^ ^)^). After study completion, data were presented to each tribe at community advisory board meetings, tribal council meetings and to tribal clinic staff, depending on the preference of each community. Summary data were also provided as a written report and in small pamphlets for community distribution. In addition, each participating family was provided with a letter summarizing their individual results and the results for their child along with information for follow-up, if desired.

## Results

### Study sample and intervention delivery

In total, 150 child–caregiver dyads completed the intervention in the mentored (*n* 67) and mailed-only (*n* 83) groups. Participants were primarily AI (>90 % for both adults and children). In our experience, some adults may identify their child as AI but may not identify themselves as such; however, all participants were living on or near reservation and were recruited from tribal areas, indicating they accessed some type of tribal services. Approximately 85 % of caregivers were the mother of the participating child (Table 1).

**Table 1:** Adult and child baseline demographic information; Healthy Children, Strong Families intervention among American Indian families with young children (2–5-year-olds), Wisconsin, USA

	Mailed group (*n* 83)		Mentored group (*n* 67)		Total (*n* 150)	
	Mean or *n*	sd or %	Mean or *n*	sd or %	Mean or *n*	sd or %
Adult						
Age (years), mean and sd	31·8	8·9	32·9	8·0	32·3	8·5
Gender, female	81	97·6	64	95·5	145	96·7
Ethnicity						
American Indian	76	91·6	64	95·5	140	93·3
White	6	7·2	3	4·5	9	6·0
Unknown	1	1·2	0	0·0	1	0·7
Relationship						
Mother	70	84·3	58	86·6	128	85·3
Father	1	1·2	2	3·0	3	2·0
Grandparent/other	12	14·5	7	6·0	19	12·7
Educational level						
High school or less	16	19·3	15	19·7	31	20·7
Some college	30	36·1	24	35·8	54	36·0
Completed college and beyond	22	26·5	16	23·9	38	25·3
Unknown	15	18·1	12	17·9	27	18·0
BMI (kg/m^2^), mean and sd	32	9·1	33	7·8	32	8·5
Current smoker (yes)	48	57·8	34	50·7	82	54·7
Child						
Age (years), mean and sd	4·0	0·9	4·0	0·9	4·0	0·9
Gender, female	36	43·4	34	50·7	70	46·7
Ethnicity						
American Indian	77	92·8	61	91·0	138	92·0
White	2	2·4	2	3·0	4	2·7
Other	3	3·6	2	3·0	5	2·3
Unknown	1	1·2	2	3·0	3	2·0
WIC participation, yes	63	75·9	47	70·1	110	73·3

WIC, Special Supplemental Nutrition Assistance Program for Women, Infants, and Children.

### Outcomes by arm

There was no effect of toolkit delivery method on primary or secondary outcomes (Table 2). When time between measures and baseline BMI was controlled for, the effect of home visiting accounted for none of the variance in child BMI *Z*-scores and <1 % in adult BMI. Because there was no effect of delivery method, study arms were combined for further analyses to test the overall efficacy of the toolkit, as both groups received the identical toolkit curriculum.

**Table 2:** Adult and child outcomes by study arm after Year 1 (post); Healthy Children, Strong Families intervention among American Indian families with young children (2–5-year-olds), Wisconsin, USA

	Mailed group				Mentored group				
	Baseline		Post		Baseline		Post		
	Mean	sd	Mean	sd	Mean	sd	Mean	sd	*P* value for mentor effect*
Adult									
BMI (kg/m^2^)†	31·3	8·2	30·8	8·1	33·0	7·3	32·7	7·0	0·495
Television on (min)	415·0	304·0	374·0	317·0	454·0	337·0	416·0	362·0	0·945
Television watching (min)	123·0	83·0	98·0	91·0	127·0	80·0	98·0	85·0	0·810
Activity count (accelerometer)	48·9	25·2	44·1	19·3	43·7	20·0	43·2	19·6	0·467
Sedentary time (%)	73·0	6·6	74·5	7·1	72·9	7·8	74·1	8·0	0·888
Fruit/vegetable intake (servings/d)	1·8	1·2	2·0	1·3	1·8	1·2	2·1	1·3	0·798
Soda/sugar intake (servings/d)	2·2	2·1	2·1	2·0	1·8	1·5	1·4	1·4	0·520
Activity change efficacy score	15·9	3·9	19·2	3·7	15·6	4·4	17·2	3·9	0·343
Nutrition change efficacy score	12·4	3·1	14·1	2·2	12·2	3·0	14·0	2·2	0·886
Physical Health score	49·5	8·8	49·4	9·3	48·3	9·7	50·7	7·8	0·182
Mental Health score	43·2	12·1	46·0	9·2	41·3	12·3	46·1	9·2	0·378
Child									
BMI (kg/m^2^)	17·5	2·5	18·0	3·2	17·3	1·6	17·9	2·5	0·701
BMI *Z*-score	1·1	1·2	1·2	1·1	1·1	1·0	1·2	1·0	0·937
BMI (percentile)	75·5	23·0	79·0	20·0	78·8	20·7	82·0	18·8	0·913
Television on (min)	413·0	282·0	333·0	270·0	460·0	347·0	443·0	379·0	0·270
Television watching (min)	119·0	84·0	98·0	69·0	113·0	76·0	107·0	67·0	0·378
Activity count (accelerometer)	85·0	31·0	94·7	38·0	94·7	35·0	100·0	31·0	0·656
Sedentary time (%)	61·5	8·1	62·8	7·4	60·5	7·1	58·6	7·3	0·129
Fruit/vegatable intake (servings/d)	1·2	0·9	1·6	1·0	1·4	1·0	1·7	1·2	0·527
Soda/sugar intake (servings/d)	0·8	0·8	0·9	1·0	0·9	0·8	0·9	0·9	0·142

* Time×treatment interaction effect assessed by repeated-measures ANOVA with study arm (mailed toolkit only *v*. mentor-delivered toolkit) as between-subjects factor.

† Participants who were pregnant for either measurement were excluded from the analyses.

### Outcomes combined

**Fig. 2:**
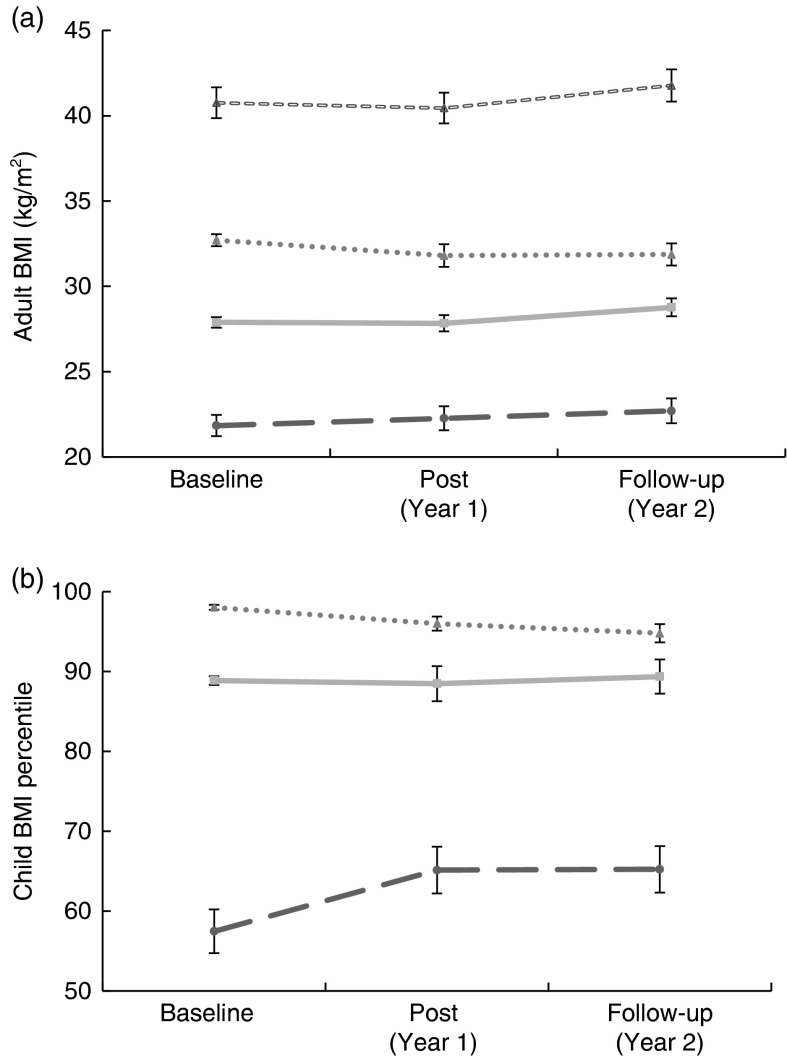
Adult BMI (a) and child BMI percentile (b) by weight status (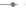, normal weight; 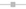, overweight; 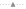, obese class I (adults)/obese (children); 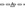, obese class II (adults only)) at baseline, post-intervention (Year 1) and follow-up (Year 2) for combined study arms (mailed group plus mentored group); Healthy Children, Strong Families intervention among American Indian families with young children (2–5-year-olds), Wisconsin, USA. Data are presented as means with their standard errors represented by vertical bars

Findings for the combined study groups are shown in Table 3. We noted a trend for a decrease in BMI and weight (in kilograms) in the combined adult group (i.e. all participants pre- to post-testing; *P*=0·09 for both). When compared with an age- and gender-matched random tribal clinic sample, the interaction (time×BMI or time×weight) was significant for both BMI (*P*=0·01) and weight change (*P*=0·01) over Year 1 between study participants (−0·38 (se 2·20) kg/m^2^ and −1·02 (se 6·00) kg) and the matched clinic controls (+0·32 (se 1·90) kg/m^2^ and +0·86 (se 5·03) kg). However, the decrease in BMI in the adult study participants rebounded when measured after maintenance Year 2 (Fig. 2(a)). When the children were analysed by weight status, we demonstrated a significant time×weight status interaction for BMI percentile from baseline through Year 2 (*P*=0·02, Fig. 2(b)). Obese children showed a reduction in BMI percentile compared with normal-weight children at Year 1 (change=−1·8 (se 3·8) % and 7·5 (se 19·2) %, respectively), while overweight children remained relatively stable (change=−0·3 (se 1·9)%). During the maintenance year, normal-weight and overweight children remained stable (change=0·09 (se 15·3) % and 0·8 (se 6·1) %, respectively) compared with a moderate decrease in weight for obese children (change=−1·2 (se 4·6) %).

**Table 3:** Adult and child outcomes for combined study arms (mailed group + mentored group) after Year 1 (post); Healthy Children, Strong Families intervention among American Indian families with young children (2–5-year-olds), Wisconsin, USA

	Baseline		Post		
	Mean	sd	Mean	sd	*P* value*
Adult					
BMI (kg/m^2^)†	32·0	7·9	31·6	7·7	0·090
Television on (min)	431·0	318·0	392·0	336·0	0·136
Television watching (min)	125·0	82·0	98·0	88·0	<0·01
Activity count (accelerometer)	46·7	23·0	43·7	19·0	0·236
Sedentary time (%)	72·9	7·1	74·3	7·4	0·182
Fruit/vegetable intake (servings/d)	1·8	1·2	2·0	1·3	0·177
Soda/sugar intake (servings/d)	2·0	1·9	1·8	1·8	0·135
Activity change efficacy score	15·8	4·1	18·4	10·1	<0·01
Nutrition change efficacy score	12·3	3·0	14·0	2·4	<0·001
Physical Health score	49·0	9·1	49·9	8·7	0·337
Mental Health score	42·5	12·1	46·0	9·7	<0·01
Child					
BMI (kg/m^2^)	17·4	2·2	17·9	3·0	<0·01
BMI *Z*-score	1·1	1·1	1·2	1·0	0·035
BMI (percentile)	76·8	22·0	80·1	19·6	0·020
Television on (min)	434·0	311·0	381·0	325·0	0·061
Television watching (min)	116·0	80·0	102·0	68·0	<0·05
Activity count (accelerometer)	89·4	32·7	97·1	31·2	0·128
Sedentary time (%)	61·1	7·6	60·9	7·6	0·898
Fruit/vegetable intake (servings/d)	1·3	0·9	1·6	1·1	<0·01
Soda/sugar intake (servings/d)	0·9	0·8	0·9	0·9	0·961

The mailed and mentored groups were combined for these analyses. Both groups received the exact same intervention materials: one group received the materials through the mail, while the second group received the materials during an in-home mentoring visit.

* Time effect measured by repeated-measures ANOVA.

† Participants who were pregnant for either measurement were excluded from the analyses.

For secondary outcomes, both children and caregivers reported significant decreases in TV watched by 27 (sd 89) min for adults and 15 (sd 79) min for children (*P*=0·002 and *P*=0·05, respectively). Children significantly increased servings of fruits and vegetables (1·3 (sd 1·0) to 1·6 (sd 1·1) servings/d, *P*=0·006), but the increase in adults was not significant. No changes in added sugar/sweetened-beverage consumption were detected. Adults reported significant improvements in mental well-being assessed by the SF-12 (*P*=0·015), but no changes were observed in perceived physical health. Moreover, adults reported improvement in both nutrition-related (*P*<0·001) and physical activity-related (*P*=0·006) self-efficacy.

### Focus group testing

Results from five focus groups indicated great acceptance of the toolkit and participants reported incorporating new healthy behaviour strategies (Table 4). A widely reported benefit of participation was increased family time through walks, active play, reading with their children, and preparing and eating family meals. Barriers to adopting healthy lifestyle behaviours included lack of social support and environmental barriers (e.g. perceived high cost of healthy foods, lack of time to practise healthy behaviours). Participants frequently mentioned that their child became a ‘change agent’ by refocusing their families on healthy behaviours.

**Table 4:** Sample focus group thematic content and adult participant quotations; Healthy Children, Strong Families intervention among American Indian families with young children (2–5-year-olds), Wisconsin, USA

Major theme	Participant comments
Increased family time	‘No more eating in their rooms. We’ve been trying to sit at the table and just talk. It’s fun to just relax with no TV and catch up with your kids.’
	‘It creates opportunities to spend more time together. My kids love it. It gets difficult to make time, so when projects come in the mail, they want to do it, even the older kids who are 8 and 11.’
	‘My daughter loved the books. We got to read them at nighttime, and it was a healthy book, so it was a new way and fun way for her to learn about healthy habits.’
Child as change agent	‘My daughter would share everything we learned, like when we’d go to my mom’s, she’d say: “You guys really should be drinking water.” So, it helped that she was learning so much, because she was sharing the information.’
	‘I don’t think my son will let me forget. He’s the one who started to bring me back after I floundered and did whatever I wanted to.’
Increased physical activity	‘I never wanted to do anything. Now I’m getting to the point where I’m trying to tell them, ”Let’s get up and go.” I’m getting more energy.’
	‘Thanks to the program, we are trying to get more involved with them during their playtime outside.’
Increased knowledge and efficacy for change	‘It gave me extra encouragement to learn and try to improve our eating habits. I read more articles and got back to reading labels carefully for ingredients and amounts. I liked the books, games, and activity ideas to use to provide fun alternatives to watching too much TV.’
	‘The lessons are very helpful. It’s like having parenting assistance, and when we get stuff in the mail it helps me set the pace for the month.’
	‘We are more aware of what we’re eating and drinking. We look at the ingredients and I even have the kids help me read the labels.’

TV, television.

## Discussion

To our knowledge, HCSF is the first intervention in AI communities to examine healthy lifestyle changes simultaneously in caregivers and young children. We did not demonstrate an effect of toolkit delivery method on our outcomes. However, when the study arms were combined to assess the effects of the HCSF toolkit, we observed decreased mean BMI percentiles in overweight and obese children post-intervention and the weight trajectory of adult participants was improved compared with a random clinic control sample. We demonstrated significantly increased fruit and vegetable consumption among children and reduced screen time in both adults and children but no changes in activity or sedentary time measured objectively via accelerometry.

The HCSF curriculum was designed to target four primary areas known to be related to obesity: (i) fruit/vegetable consumption; (ii) intake of empty calories; (iii) physical activity; and (iv) screen time. Of note, this curriculum was designed to promote family wellness rather than weight loss. As such, participants from all weight classifications were included rather than targeting children or adults who were already overweight/obese^(^ ^24^ ^)^. Although our study adults did not experience significant weight loss, their weight trajectory was significantly improved compared with a matched tribal clinic control sample. Given that the average weight gain for adults in the USA is 0·5–1·0 kg/year^(^ ^25^ ^)^ (which aligns with the annual weight gain observed in our clinic sample of +0·86 kg), this improvement in the HCSF participants’ weight trajectory may be clinically significant. Moreover, the weight loss demonstrated in the obese children post-intervention and sustained through the maintenance year is particularly encouraging, as weight-loss rebound has been well documented after clinical interventions targeting lifestyle changes^(^ ^26^ ^–^ ^28^ ^)^. The observed improvements in weight-related behaviours (e.g. increased fruit and vegetable consumption, decreased screen time) may support these findings.

Our findings were demonstrated in an age group (2–5 years) for whom relatively few wellness interventions have been tested, particularly over a long duration^(^ ^7^ ^)^. In addition, most previous approaches did not include a parental component (e.g. adult outcome measures or interventions designed to include adults). For HCSF, health-related outcomes for both adults and children were primary targets in recognition of the critical importance of the family unit and caregiver engagement in sustained behaviour change. Our approach included adults in both the outcome measures and in the execution of the intervention, as toolkit lessons were designed to increase interaction within families. We believe the inclusion of both adults and children strengthened the delivery and efficacy of the toolkit curriculum, as improvements in health behaviours were observed in both adults and children. An unanticipated qualitative outcome was the report of increased family time, specifically around family meals and reading together. Although this change cannot be quantified, increased family time has been shown to relate to positive behavioural^(^ ^29^ ^)^ and obesity-related outcomes^(^ ^30^ ^)^ in older children and represents an area for further inquiry.

The HCSF toolkit intervention was designed for use in participants’ homes, which represents another strength of the current study. The home environment is critical to young children, as the majority of health behaviour modelling occurs there^(^ ^31^ ^,^ ^32^ ^)^. Of the few interventions conducted in the 2–5 years age group, most were delivered in school or child-care settings^(^ ^12^ ^)^. While several of these school- or centre-based interventions included a parental component (e.g. newsletters), they were not designed to increase parental self-efficacy for the application of health-related behaviours within the home. Our study demonstrated the efficacy of a healthy lifestyles toolkit within a home-based setting delivered in a culturally sensitive way that was supported by communities. Another programme reported similar benefits of involving Head Start centres in interventions to prevent childhood obesity in AI and Hispanic communities^(^ ^33^ ^)^, highlighting the importance of community context in intervention design and delivery.

The present study has several limitations to note. First, significant dropout occurred with the study participants, particularly within the mentored group. This dropout may be related to the participant burden inherent in scheduling a regular time for a mentor to visit their home and issues around the trust necessary to allow another person into a personal space (including mentor turnover). In addition, this pilot investigation included a relatively small sample size. Moreover, we were unable to address other related factors, such as stress, lifestyle disruptions caused by shift-work jobs, poverty, historical trauma and substance abuse, that contribute to the complexity of the health environment experienced by these families.

Our HCSF study revealed some promising health-related changes resulting from a family-focused obesity prevention toolkit. In addition, we noted substantial interest and support within the participating tribal communities. All four communities have continued to address obesity prevention since HCSF ended, with several securing their own external funding for projects. We believe the ongoing support will substantially impact future child outcomes within these communities. Further testing of an expanded mailed-only intervention is currently in the field at five rural and urban AI sites nationally. If successful, these interventions should be paired with multilevel community-based interventions in pre-schools/schools, worksites and the built environment to enable families to make healthy choices more easily, particularly in underserved communities.
